# Interoception Primes Emotional Processing: Multimodal Evidence from Neurodegeneration

**DOI:** 10.1523/JNEUROSCI.2578-20.2021

**Published:** 2021-05-12

**Authors:** Paula C. Salamone, Agustina Legaz, Lucas Sedeño, Sebastián Moguilner, Matías Fraile-Vazquez, Cecilia Gonzalez Campo, Sol Fittipaldi, Adrián Yoris, Magdalena Miranda, Agustina Birba, Agostina Galiani, Sofía Abrevaya, Alejandra Neely, Miguel Martorell Caro, Florencia Alifano, Roque Villagra, Florencia Anunziata, Maira Okada de Oliveira, Ricardo M. Pautassi, Andrea Slachevsky, Cecilia Serrano, Adolfo M. García, Agustín Ibañez

**Affiliations:** ^1^Cognitive Neuroscience Center, Universidad de San Andrés, Buenos Aires, Argentina; ^2^National Scientific and Technical Research Council (CONICET), Buenos Aires, Argentina; ^3^Facultad de Psicología, Universidad Nacional de Córdoba, Córdoba, Argentina; ^4^Global Brain Health Institute, University of California-San Francisco, San Francisco, California, and Trinity College Dublin, Dublin, Ireland; ^5^Nuclear Medicine School Foundation, National Commission of Atomic Energy, Mendoza, Argentina; ^6^Institute of Cognitive and Translational Neuroscience, INECO Foundation, Favaloro University, CONICET, Buenos Aires, Argentina; ^7^Latin American Brain Health (BrainLat), Universidad Adolfo Ibáñez, Santiago, Chile; ^8^Memory and Neuropsychiatric Clinic, Neurology Department, Hospital del Salvador, SSMO & Faculty of Medicine, University of Chile, Santiago, Chile; ^9^Instituto de Investigación Médica M. y M. Ferreyra, INIMEC-CONICET-UNC, Córdoba, Argentina; ^10^Department of Neurology, Faculdade de Medicina FMUSP, Universidade de Sao Paulo, Sao Paulo, SP Brazil; ^11^Department of Neurology, Hospital Santa Marcelina, Sao Paulo, SP Brazil; ^12^Gerosciences Center for Brain Health and Metabolism, Santiago, Chile; ^13^Neuropsychology and Clinical Neuroscience Laboratory, Physiopathology Department, ICBM, Neurosciences Department, Faculty of Medicine, University of Chile, Santiago, Chile; ^14^Servicio de Neurología, Departamento de Medicina, Clínica Alemana-Universidad del Desarrollo, Santiago, Chile; ^15^Neurología Cognitiva, Hospital Cesar Milstein, Buenos Aires, Argentina; ^16^Faculty of Education, National University of Cuyo, Mendoza, M5502JMA, Argentina; ^17^Departamento de Lingüística y Literatura, Facultad de Humanidades, Universidad de Santiago de Chile, Santiago, Chile

**Keywords:** Alzheimer's disease, behavioral variant frontotemporal dementia, emotion, interoception, Parkinson's disease, priming

## Abstract

Recent frameworks in cognitive neuroscience and behavioral neurology underscore interoceptive priors as core modulators of negative emotions. However, the field lacks experimental designs manipulating the priming of emotions via interoception and exploring their multimodal signatures in neurodegenerative models. Here, we designed a novel task that involves interoceptive and control-exteroceptive priming conditions followed by post-interoception and post-exteroception facial emotion recognition (FER). We recruited 114 participants, including healthy controls (HCs) as well as patients with behavioral variant frontotemporal dementia (bvFTD), Parkinson's disease (PD), and Alzheimer's disease (AD). We measured online EEG modulations of the heart-evoked potential (HEP), and associations with both brain structural and resting-state functional connectivity patterns. Behaviorally, post-interoception negative FER was enhanced in HCs but selectively disrupted in bvFTD and PD, with AD presenting generalized disruptions across emotion types. Only bvFTD presented impaired interoceptive accuracy. Increased HEP modulations during post-interoception negative FER was observed in HCs and AD, but not in bvFTD or PD patients. Across all groups, post-interoception negative FER correlated with the volume of the insula and the ACC. Also, negative FER was associated with functional connectivity along the (a) salience network in the post-interoception condition, and along the (b) executive network in the post-exteroception condition. These patterns were selectively disrupted in bvFTD (a) and PD (b), respectively. Our approach underscores the multidimensional impact of interoception on emotion, while revealing a specific pathophysiological marker of bvFTD. These findings inform a promising theoretical and clinical agenda in the fields of nteroception, emotion, allostasis, and neurodegeneration.

**SIGNIFICANCE STATEMENT** We examined whether and how emotions are primed by interoceptive states combining multimodal measures in healthy controls and neurodegenerative models. In controls, negative emotion recognition and ongoing HEP modulations were increased after interoception. These patterns were selectively disrupted in patients with atrophy across key interoceptive-emotional regions (e.g., the insula and the cingulate in frontotemporal dementia, frontostriatal networks in Parkinson's disease), whereas persons with Alzheimer's disease presented generalized emotional processing abnormalities with preserved interoceptive mechanisms. The integration of both domains was associated with the volume and connectivity (salience network) of canonical interoceptive-emotional hubs, critically involving the insula and the anterior cingulate. Our study reveals multimodal markers of interoceptive-emotional priming, laying the groundwork for new agendas in cognitive neuroscience and behavioral neurology.

## Introduction

Interoception (Intero, the sensing of visceral signals) seems intertwined with facial emotion recognition (FER), especially negative emotions (Barrett and Simmons, 2015; Garfinkel and Critchley, 2016). Still, beyond metanalytical findings (Adolfi et al., 2017) and partial associations (Pollatos and Schandry, 2008; Terasawa et al., 2014), evidence of how Intero primes FER is limited in healthy populations and absent in neurodegenerative conditions. Here, we combined neurophysiological, neuroanatomical, and functional connectivity (FC) measures with a novel task capturing interoceptive effects on FER in four groups: healthy controls (HCs) and patients with behavioral variant frontotemporal dementia (bvFTD), Parkinson's disease (PD), and Alzheimer's disease (AD).

Behavioral evidence from HCs suggests that Intero increases sensitivity to negative FER (Terasawa et al., 2014; Critchley and Garfinkel, 2017). Alterations in both domains are systematic in bvFTD (Piguet et al., 2011; Kumfor et al., 2013; Van den Stock and Kumfor, 2017; Ibáñez, 2019) and less marked in PD (Enrici et al., 2015; Argaud et al., 2018), contrasting with the interoceptive pattern (García-Cordero et al., 2016) and generalized emotional deficits (Klein-Koerkamp et al., 2012) observed in AD.

Electrophysiologically, an interoceptive frontal cortical marker (heart-evoked potential [HEP]) has been linked to emotional processing in HCs (Couto et al., 2015). Conversely, HEP modulations during Intero are abnormal in bvFTD, dependent on fronto-temporo-insular integrity in AD (García-Cordero et al., 2016), and uncharted in PD, although this disorder involves disruptions of fronto-central potentials during emotional facial processing (Garrido-Vásquez et al., 2016).

Regarding neuroimaging, evidence highlights the insula and the ACC as key regions integrating interoceptive and emotional processes (Adolfi et al., 2017; J. Kim et al., 2019). Such convergence is supported by the salience network (SN), which encompasses the abovementioned areas and is involved in processing internal events (Seeley et al., 2007), including interoceptive and affective signals (Uddin, 2015). This cross-modal role differentiates the SN from other networks subserving emotional (Lindquist and Barrett, 2012) and exteroceptive (Sridharan et al., 2008) processes, such as the frontoparietal executive network (EN). Suggestively, the SN is distinctively affected in bvFTD relative to HCs and AD patients (Zhou et al., 2010; Seeley, 2019), and its connectivity with striatal hubs correlates with disease severity in PD (Putcha et al., 2015).

**Figure 1.**
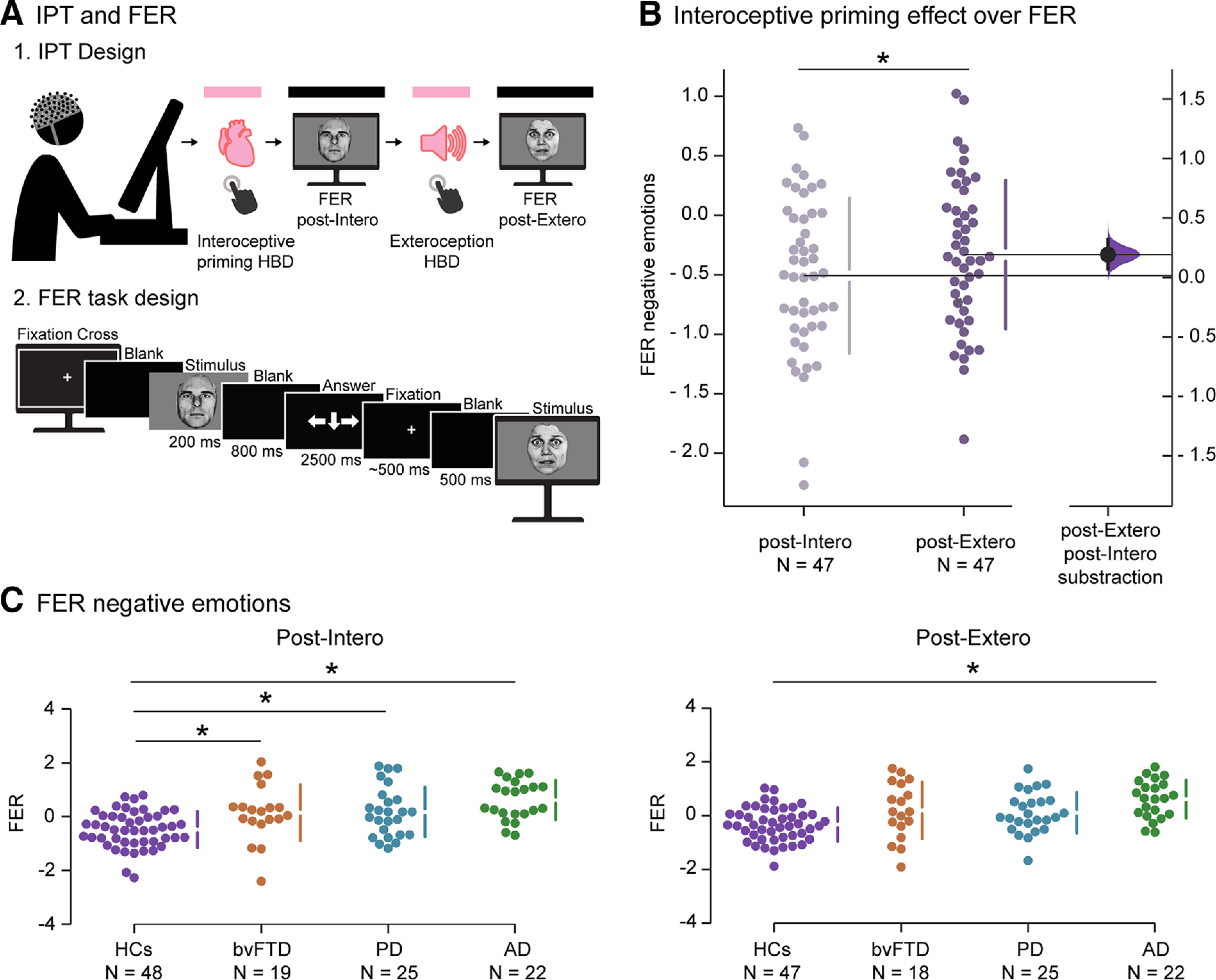
Task and behavioral results. ***A***, IPT and FER task. ***A1***, Task design. Participants performed the task facing a computer, with hd-EEG recordings obtained. The priming phase included an interoceptive and an exteroceptive condition (pink, counterbalanced), followed by the FER phase (black). Each priming condition was performed twice. For tapping-priming results, see Table 10. ***A2***, FER task design. Illustration of timing and sequence of stimuli on screen. Answer options: negative, neutral, or positive (←, ↓, and →, respectively). ***B***, Interoceptive priming effect over FER. Results of FER negative emotions comparing post-Intero and post-Extero effects in HCs. For results of interoceptive-priming effects on FER of all emotion types, see Figure 2. ***C***, Recognition of negative emotions in the post-Intero and post-Extero conditions. We compared the behavioral performance of HCs and patient group via one-way ANOVA and Tukey *post hoc* comparisons using the normalized IES. For details of IES non-normal distribution, see Table 8. For details on FER results, see Table 11. Dot-plots represent results for HCs (purple), bvFTD (orange), PD (light blue), and AD (green) participants. Vertical gapped lines indicate mean (gap) and SD (lines). *Significant difference.

Yet, such links have been mainly captured through partial associations between separate interoceptive and emotional tasks. Therefore, they fail to reveal whether Intero can directly influence emotional processing at different behavioral and neurocognitive levels, limiting theoretical and clinical developments (Van den Stock and Kumfor, 2017). This can be achieved through priming paradigms, which have illuminated the impact of Intero on other social cognition processes (Ernst et al., 2013), and of cardiac dynamics on negative FER (Pezzulo et al., 2018). Here, we developed an interoceptive priming task (IPT) comprising (1) a priming phase, including counterbalanced interoceptive and control-exteroceptive conditions; and (2) an FER phase after each priming condition (post-Intero and post-exteroception [Extero]) (see Fig. 1*A*). This design can reveal novel, direct links between Intero and emotion.

We advanced three sets of hypotheses. First, we predicted that HCs would show enhanced negative FER in the post-Intero condition, and that such effect would be selectively disrupted in bvFTD and PD patients relative to HCs, contrasting with generalized FER alterations in AD. For bvFTD, such disruptions would be accompanied by impaired interoceptive accuracy. Second, we hypothesized that HEP modulations would significantly increase during post-Intero-negative FER in HCs, but not in bvFTD and PD. Third, across all groups, we expected that post-Intero-negative FER would correlate with the volume of the insula and the ACC as well as connectivity along networks involving such hubs (the SN). Instead, post-Extero-negative FER was expected to correlate with EN connectivity. Moreover, we predicted that associations between post-Intero-negative FER and SN connectivity would be abolished in bvFTD, and that no other group would show this specific pattern across networks and conditions. By testing these hypotheses, we aim to understand how interoceptive and emotional processes converge in the brain.

## Materials and Methods

### 

#### Participants

To determine the sample size required for our study, we ran a power estimation analysis on G*Power 3.1 (Faul et al., 2007). Given our statistical design (one-way ANOVA among groups and *post hoc* comparisons between HCs and each patient group), we considered the following parameters: a large effect size of *f* = 0.40, an α level of *p* = 0.05, and a power of 0.80, higher than the median of powers analyzed in previous studies in the area for detecting large effect sizes (Szucs and Ioannidis, 2017). This analysis showed that a total sample size of 76 is adequate to detect the estimated effects. The study comprised 114 participants, reaching a power of 0.95. All were part of an ongoing multicenter protocol, which follows recommendations for similar designs (Sedeño et al., 2017; Moguilner et al., 2018, 2021; Bachli et al., 2020; Ibáñez et al., 2021a,b). We recruited 48 HCs with no history of psychiatric or neurologic disease, 19 patients fulfilling revised criteria for bvFTD (Rascovsky et al., 2011), 25 PD patients diagnosed in accordance with the United Kingdom Parkinson's Disease Society Brain Bank criteria (Hughes et al., 1992), and 22 AD patients who fulfilled international National Institute of Neurological and Communicative Disorders and Stroke–Alzheimer's Disease and Related Disorders Association criteria (Dubois et al., 2007; McKhann et al., 2011). Patients did not exhibit specific psychiatric disorders or primary language deficits. Samples were recruited from three international clinics. Each patient sample was matched on gender, age, and education with HCs (Table 1). The patients' clinical diagnosis was established in each center through an extensive neurologic, neuropsychiatric, and neuropsychological examination (Table 1) and revised by neurodegenerative disease experts, as done previously (Baez et al., 2014; Melloni et al., 2016; Sedeño et al., 2017; Donnelly-Kehoe et al., 2019).

**Table 1.** Demographic and neuropsychological results*^a^*

Variable	HCs	bvFTD	PD	AD	Statistical results	*Post hoc*
Demographics						
Sex	27:21	6:13	12:13	11:11	χ^2^ = 3.33, *p* = 0.34	
Age**	71.58 (6.09)	68.47 (10.47)	73.08 (6.98)	74.64 (5.34)	*F* = 2.85, *p* = 0.04*, ηp^2^ = 0.07	HCs-bvFTD: *p* = 0.36 HCs-PD: *p* = 0.82 HCs-AD: *p* = 0.33
Education	14.12 (3.75)	14.53 (5.36)	11.84 (4.44)	11.59 (3.81)	*F* = 3.33, *p* = 0.02*, ηp^2^ = 0.08	HCs-bvFTD: *p* = 0.98 HCs-PD: *p* = 0.13 HCs-AD: *p* = 0.09
Cognitive screening results						
MoCA**	25.3 (2.9)	19.95 (5.24)	21.04 (4.29)	16.27 (4.71)	*F* = 27.34, *p* < 0.001*, ηp^2^ = 0.42	HCs-bvFTD: *p* < 0.001* HCs-PD: *p* < 0.001* HCs-AD *p* < 0.001*
IFS**	21.01 (3.31)	17.11 (5.82)	19.16 (4.59)	14.45 (4.76)	*F* = 12.32, *p* < 0.001*, ηp^2^ = 0.25	HCs-bvFTD: *p* = 0.007* HCs-PD: *p* = 0.44 HCs-AD: *p* < 0.001*

*^a^*Data are mean (SD). Demographic and clinical data were assessed through ANOVAs, except for sex, which was analyzed via Pearson's χ^2^ test. Effect sizes were calculated through ηp^2^. MoCA, Montreal Cognitive Assessment (Nasreddine et al., 2005); IFS, Ineco Frontal Screening.

*Significant difference.

**Variable with significant differences (*p* < 0.05) between patient groups, precluding comparisons between them in our target measures.

As expected, whole-brain gray matter (GM) analyses (*p* < 0.05, familywise error [FWE] correction, extent threshold = 50 voxels) revealed fronto-temporo-insular atrophy in bvFTD patients (Whitwell et al., 2009; Piguet et al., 2011; Ibáñez and Manes, 2012), no GM atrophy in PD patients (Huber et al., 1989; Schulz et al., 1999; Price et al., 2004), and a predominantly bilateral temporal pattern (with smaller frontal compromise) in AD patients (Du et al., 2007; Pini et al., 2016) (Table 2). bvFTD patients were functionally impaired and exhibited prominent changes in personality and social behavior, as verified by caregivers. PD patients were medicated with antiparkinsonian therapy and evaluated during “on” phase. Finally, AD patients were also functionally impaired as verified by caregivers. Cognitive impairments were assessed in all patients with the Montreal Cognitive Assessment (Nasreddine et al., 2005) and a clinical interview with experts. Further clinical and behavioral features of patients are described in Table 3.

**Table 2.** GM atrophy patterns in patients*^a^*

Region	Cluster		Peak		MNI coordinates	
	No. of voxels	*p* (FWE)	*t*	*x*	*y*	*z*
bvFTD atrophy						
Middle temporal R	869	0.008	5.03	42	−27	−8
Inferior temporal R			4.68	51	−18	−21
Middle temporal R			4.57	51	−29	−11
Middle temporal pole R	446	0.0178	4.7	44	6	−32
Inferior orbital frontal R	661	0.011	4.56	29	27	−9
Inferior orbital frontal R			4.25	38	18	−21
Insula L	124	0.0474	4.28	−33	21	8
AD atrophy						
Hippocampus R	24779	0.0002	6.89	32	−9	−18
Hippocampus L			6.87	−24	−8	−20
Amygdala L			6.77	−23	2	−21
Middle temporal R	294	0.0216	4.83	53	−30	−12
Middle frontal L	186	0.0334	4.54	−41	48	5
Inferior orbital frontal L			4.23	−39	41	−6
Insula L	333	0.0184	4.49	−36	14	2
Insula L			4.28	−26	11	14
Insula L			4.2	−26	20	−3

*^a^*Atrophy in each patient group was calculated via voxel-based morphometry, based on *w*-score maps of the normalized and smoothed DARTEL outputs. We ran two-sample *t* tests between patients and HCs using the statistical nonparametric mapping (SnPM13; http://www.fil.ion.ucl.ac.uk/spm/snpm) toolbox for SPM12, based on 5000 random permutations, covarying for total intracranial volume. Significance was set to *p* < 0.05 (extent threshold = 50 voxels), with FWE correction. bvFTD patients showed atrophy in the right inferior orbitofrontal gyrus, the right inferior and middle temporal gyri, the right middle temporal pole, and the left insula. AD patients showed atrophy along the bilateral hippocampus, the right middle temporal gyrus, the left middle frontal and inferior orbitofrontal gyri, the left insula, and the left amygdala. No atrophy was found in PD patients. Results are presented on MNI space using the AAL atlas, in the neurologic convention. R, Right; L, left.

**Table 3.** Clinical and behavioral features of patients*^a^*

	bvFTD	PD	AD
FAQ^b^* (Pfeffer et al., 1982)	10.42 (7.36)	4.46 (5.65)	15.91 (7.56)
FRS^c^* (Mioshi et al., 2010)	−0.05 (1.49)	1.37 (1.22)	−0.98 (1.47)

*^a^*Data are mean (SD).

*^b^*FAQ, Functional Activities Questionnaire, a 10 item rating scale that measures instrumental activities of daily living (e.g., preparing meals and personal finance) (Pfeffer et al., 1982). A score >9 suggests a possible impaired function and possible cognitive impairment.

*^c^*FRS, Frontotemporal Dementia Rating Scale, a 30 item scale that evaluates severity in patients with dementia (Mioshi et al., 2010). Scores from 1.92 to −2.58 indicate a moderate/severe disease stage; scores from −2.58 to −6.66 indicate a very severe/profound disease stage.

*Variable with significant differences (*p* < 0.05) between patient groups, precluding comparisons between them in our target measures.

The institutional ethics committees of each recruitment center approved the study protocol. All participants (or their caregivers) provided signed informed consent in accordance with the Declaration of Helsinki.

#### Experimental design

##### Behavioral task

The IPT included two phases: (1) a tapping-priming phase, in which subjects have to follow their own heartbeats [interoceptive condition (Intero)] or a recorded one [control exteroceptive condition (Extero)]; and (2) a subsequent FER phase, in which participants were requested to identify the emotion of static faces. The tapping-priming phase consisted of four blocks of 2 min each, with two blocks per condition (Intero and Extero), which were always counterbalanced across participants. The order of presentation was also counterbalanced per subject per group, so half of them started with Intero and the other half started with Extero. Immediately after each tapping-priming block, a block of the FER phase was administered, resulting in two post-Intero FER blocks and two post-Extero FER blocks (four FER blocks in total). Before the test, participants had a 2 min tapping-priming practice phase and an FER practice phase. High-density EEG (hd-EEG) recordings were obtained during the FER blocks for each participant to evaluate the impact of interoceptive priming on the HEP correlates of FER.

For the tapping-priming phase, we used a validated heartbeat detection (HBD) task (Canales-Johnson et al., 2015; García-Cordero et al., 2016; Yoris et al., 2017, 2018; Salamone et al., 2018, 2020; Abrevaya et al., 2020; Richter et al., 2021; Richter and Ibáñez, 2021). Participants were required to tap a computer keyboard along with their heartbeats (Intero) or external stimuli (Extero). Two blocks were presented for each condition. In each Intero condition, subjects were asked to follow their own heartbeats in the absence of any sensory feedback, for 2 min. This condition provides an objective measure of each participant's interoceptive accuracy (Garfinkel et al., 2015). In Extero, participants were instructed to follow an audio recording of a simulated heartbeat for 2 min, as a control measure to test the influence of noninteroceptive priming over FER. An accuracy score (*d*′) was estimated for each condition per subject based on HBD outcomes via signal detection theory (Macmillan and Creelman, 2004; Thomson and Kristan, 2005; Gonzalez Campo et al., 2020). This approach yields robust motor-tracking HBD indexes, as it allows estimating subjects' signal-to-noise sensitivity and specificity by penalizing correct responses made by chance (Brener and Ring, 2016; Richter and Zimmer, 2018). Accordingly, it is preferable to more classical measures, such as Schandry's index (Schandry, 1981), which can be contaminated by confounds, such as heart rate (HR) estimations and total number of responses (Zamariola et al., 2018; Fittipaldi et al., 2020; Richter and Zimmer, 2020).

The FER phase was based on Ekman and Friesen's images (Ekman and Friesen, 1971; Ekman, 1976). In each of the four FER blocks, 56 faces were presented in a different pseudorandomized order per block, for 200 ms each. The stimuli comprised 8 neutral, 16 positive (8 for happiness, 8 for surprise), and 32 negative (8 for anger, 8 for disgust, 8 for sadness, 8 for fear) faces. All images denoted their corresponding emotion with the same intensity (100%). Participants were asked to judge, as fast and accurately as possible, whether each face presented a negative, neutral, or positive emotion by pressing preassigned keyboard arrows. FER performance was quantified through the Inverse Efficiency Score (IES) (Hesse et al., 2016, 2019), a standard metric that combines reaction time (RT) and accuracy data to holistically establish weighted behavioral outcomes (Jacques and Rossion, 2007; Jacquet and Avenanti, 2015). Specifically, it is calculated by dividing the mean RT by the proportion of correct responses (Mevorach et al., 2006; Brozzoli et al., 2008), thus controlling for biases introduced by fast RTs with low accuracy and vice versa. Therefore, the higher the IES, the poorer the performance. The average IES from the two post-Intero and the two post-Extero FER blocks was obtained per subject for each emotion type (negative, positive, and neutral).

##### hd-EEG data acquisition

We obtained hd-EEG signals during the FER phase from 100 participants. These comprised 18 bvFTD, 23 PD, and 18 AD patients, each group being demographically matched with HCs (*n* = 41) (Table 4). Recordings were performed with a Biosemi Active-two 128-channel system at 1024 Hz, following standard preprocessing steps (García-Cordero et al., 2016, 2017; Yoris et al., 2017, 2018; Salamone et al., 2018), as detailed in hd-EEG preprocessing and analysis.

**Table 4.** HEP: demographic results*^a^*

Variable	HCs	bvFTD	PD	AD	Statistical results	*Post hoc*
Sex	24:20	3:12	11:10	10:9	χ^2^ = 5.77, *p* = 0.12	
Age	71.54 (6.02)	68.39 (10.77)	72.83 (7)	74.06 (5.33)	*F* = 2.10, *p* = 0.10, ηp^2^ = 0.06	HCs-bvFTD: *p* = 0.41 HCs-PD: *p* = 0.90 HCs-AD: *p* = 0.60
Education	13.8 (3.8)	15.11 (4.85)	11.48 (4.28)	11.56 (3.76)	*F* = 3.87, *p* = 0.01*, ηp^2^ = 0.10	HCs-bvFTD: *p* = 0.67 HCs-PD: *p* = 0.13 HCs-AD: *p* = 0.22

*^a^*Data are mean (SD). Demographic data were assessed through ANOVAs. Gender was analyzed with the Pearson χ^2^ test. Effect sizes were calculated through ηp^2^.

*Significant difference.

##### MRI/fMRI data acquisition

MRI and fMRI acquisition and preprocessing steps are reported as recommended by the Organization for Human Brain Mapping (Nichols et al., 2017; Poldrack et al., 2017). In each center, following standard protocols (García-Cordero et al., 2016; Gonzalez Campo et al., 2020), we obtained 3D volumetric and 10-min-long resting-state MRI sequences from all participants, recordings were performed in three scanners (for details, see Table 5). For the resting-state protocol, participants were asked not to think about anything in particular, to keep their eyes closed, and to avoid moving or falling asleep. We chose the closed-eyes modality to avoid noisy signals coming from the visual cortex. Fifteen 3D volumetric images (4 HCs; 4 bvFTD; 4 PD; 3 AD) and 18 fMRI acquisition (4 HCs; 6 bvFTD; 4 PD; 4 AD) were excluded because of absence of imaging data or artifacts. These final MRI and fMRI samples were demographically matched (for details, see Table 6 and Table 7, respectively).

**Table 5.** Specific neuroimaging parameters per left*^a^*

	Parameter
left 1	3-T Phillips scanner with a standard head coil, whole-brain T1-rapid anatomic 3D gradient echo volumes were acquired parallel to the plane connecting the anterior and posterior commissures, with the following parameters: TR = 8300 ms; TE = 3800 ms; flip angle = 8º; 160 slices, matrix dimension = 224 × 224 × 160; voxel size = 1 mm × 1 mm × 1 mm. Also, functional spin echo volumes, parallel to the anterior-posterior commissures, covering the whole brain, were sequentially and ascendingly acquired with the following parameters: TR = 2640 ms; TE = 30 ms; flip angle = 90º; 49 slices, matrix dimension = 80 × 80 × 49; voxel size in plane = 3 mm × 3 mm × 3 mm; slice thickness = 3 mm; sequence duration = 10 min; number of volumes = 220.
left 2	Using a 3-T Siemens Skyra scanner with a standard head coil, we acquired whole-brain T1-rapid gradient echo volumes, parallel to the plane connecting the anterior and posterior commissures, with the following parameters: TR = 1700 ms; TE = 2000 ms; flip angle = 8º; 208 slices, matrix dimension = 224 × 224 × 208; voxel size = 1 mm × 1 mm × 1 mm. On the other hand, functional EP2D-BOLD pulse sequences, parallel to the anterior-posterior commissures, covering the whole brain, were acquired sequentially, intercalating pair-ascending first with the following fMRI parameters: TR = 2660 ms; TE = 30 ms; flip angle = 90º; 46 slices, matrix dimension = 76 × 76 × 46; voxel size in plane = 3 mm × 3 mm × 3 mm; slice thickness = 3 mm; sequence duration = 13.3 min; number of volumes = 300.
left 3	Using a 3-T Siemens Skyra scanner with a standard head coil, we acquired whole-brain T1-rapid gradient echo volumes, parallel to the plane connecting the anterior and posterior commissures, with the following parameters: TR = 2400 ms; TE = 2000 ms; flip angle = 8º; 192 slices, matrix dimension = 256 × 256 × 192; voxel size = 1 mm × 1 mm × 1 mm. Finally, functional EP2D-BOLD pulse sequences, parallel to the anterior-posterior commissures, covering the whole brain, were acquired sequentially, intercalating pair-ascending first with the following fMRI parameters: TR = 2660 ms; TE = 30 ms; flip angle = 90º; 46 slices, matrix dimension = 76 × 76 × 46; voxel size in plane = 3 mm × 3 mm × 3 mm; slice thickness = 3 mm; sequence duration = 10.5 min; number of volumes = 240.

*^a^*Following standard protocols (García-Cordero et al., 2016; Gonzalez Campo et al., 2020), we obtained 3D volumetric and 10-min-long resting-state MRI sequences from all participants; recordings were performed in three scanners.

**Table 6.** MRI-T1: demographic results*^a^*

Variable	HCs	bvFTD	PD	AD	Statistical results	*Post hoc*
Sex	24:20	3:12	11:10	10:9	χ^2^ = 5.77, *p* = 0.12	
Age	71.36 (6)	68.73 (10.71)	72.86 (7.52)	74.68 (5.32)	*F* = 2.17, *p* = 0.09, ηp^2^ = 0.06	HCs-bvFTD: *p* = 0.60 HCs-PD: *p* = 0.85 HCs-AD: *p* = 0.32
Education	14.05 (3.86)	14.73 (5.22)	11.62 (4.48)	11.16 (3.32)	*F* = 3.85, *p* = 0.01*, ηp^2^ = 0.10	HCs-bvFTD: *p* = 0.94 HCs-PD: *p* = 0.12 HCs-AD: *p* = 0.059

*^a^*Data are mean (SD). Demographic data were assessed through ANOVAs. Gender was analyzed with the Pearson χ^2^ test. Effect sizes were calculated through ηp^2^.

*Significant difference.

**Table 7.** fMRI Demographic results*^a^*

Variable	HCs	bvFTD	PD	AD	Statistical results	*Post hoc*
Sex	24:20	3:10	11:10	9:9	χ^2^ = 4.14, *p* = 0.24	
Age	71.36 (6)	68.31 (10.67)	72.86 (7.52)	74.94 (5.35)	*F* = 2.47, *p* = 0.06, ηp^2^ = 0.07	HCs-bvFTD: *p* = 0.51 HCs-PD: *p* = 0.85 HCs-AD: *p* = 0.26
Education	14.05 (3.86)	14.08 (5.3)	11.62 (4.48)	11.39 (3.26)	*F* = 2.95, *p* = 0.03*, ηp^2^ = 0.08	HCs-bvFTD: *p* = 0.99 HCs-PD: *p* = 0.12 HCs-AD: *p* = 0.10

*^a^*Data are mean (SD). Demographic data were assessed through ANOVAs. Gender was analyzed with the Pearson χ^2^ test. Effect sizes were calculated through ηp^2^.

*Significant difference.

#### Statistical analysis

##### Statistical analysis for behavioral outcomes

As in previous reports with neurodegenerative diseases (Sollberger et al., 2009, 2014; Shany-Ur et al., 2012, 2014; Chiong et al., 2016; García-Cordero et al., 2016, 2019), our hypotheses hinged on differences between each patient group and HCs. Hence, behavioral statistical analyses were performed to compare group pairs of control and patients: HCs versus bvFTD, HCs versus PD, HCs versus AD (the same approach was adopted for the HEP analyses).

For the tapping-priming phase, the *d*′ index was calculated in terms of signal detection theory, a framework that allows distinguishing ambiguous stimuli of a task as signal or noise (Macmillan and Creelman, 2004). In the HBD task, a heartbeat is considered “signal,” whereas the absence of a heartbeat is “noise.” Subjects' reactions can be classified as “yes” (tapping the keyboard) or “no” (not tapping the keyboard) (Richter and Zimmer, 2018). If a yes response occurs in a given window time-locked to the R-wave of the preceding heartbeat (signal), then it is considered correct and called “hit.” On the contrary, the absence of a response in the defined temporal window is considered a “miss.” In addition, the signal detection theory formula weighs the strategy of the subject in discriminating signal from noise, to penalize successful responses by chance (e.g., a subject who always responds yes would get all hits). Hence, a response outside the window is a “false alarm,” whereas the absence of a response outside the window is a correct rejection. To control for potential interindividual differences, the temporal extension of the window was determined for each subject according to his HR. The window was locked 750 ms after the beat for a HR <69.76; 600 ms after the beat for a HR between 69.76 and 94.25; and 400 ms after the beat for a HR >94.25 (Salamone et al., 2018). Higher values of *d*′ indicate better discrimination ability and thus better interoceptive accuracy in the interoceptive condition, and better tapping accuracy in the exteroceptive condition. We calculated *d*′ index with the following equation:

$$d\prime =\left(z\frac{\sum hits}{\sum hits+\sum miss}\right)-\left(z\frac{\sum falsealarm}{\sum falsealarm+\sum correctrejection}\right)$$

Where *z* (*p*) is the inverse normal probability corresponding to cumulative probability. To calculate the tapping index of the exteroceptive condition, we used a window between 0 and 750 ms after the recorded heartbeat. After this, to test our hypothesis of specific interoceptive accuracy disruptions in bvFTD compared with HCs, *t* tests were run to compare the IPT's *d*′ index between HCs and each patient group, using Statistica (StatSoft) software.

For the FER phase, to improve the responses' signal-to-noise ratio (avoiding responses biased by attentional distraction or technical problems in the recordings), we removed trials with RTs > 2500 ms (Pérez-Mata et al., 2012; Hatzidaki et al., 2015; Pelaez et al., 2016) and then also excluded those that fell 3 SDs away from the mean of each subjects' RT. Also, to exclude data that may not reflect our target psychological processes in both phases of the task, we discarded subjects whose mean RTs fell 3 SDs from the sample's mean in each condition (de la Fuente et al., 2019).

Given that Shapiro–Wilk's tests revealed non-normal distributions for FER indexes (Table 8), and that analyses based on non-normalized data may promote Type I and Type II errors, we normalized the IES scores using box-Cox transformation, previously applied in emotion recognition studies (Kuperman et al., 2014; Statucka and Walder, 2017) for behavioral analyses. This approach supersedes traditional normalization procedures as it fulfills the assumptions of normality, linearity, and homoscedasticity (Sakia, 1992; Osborne, 2010), improving results' generalizability and effect size precision (Osborne, 2010). Behaviorally, we predicted that HCs would show enhanced negative FER in the post-Intero condition. To test this hypothesis, FER performance was compared in HCs between conditions for emotion type via two-tailed *t* tests. Moreover, we hypothesized that such enhancement effect would be selectively disrupted in bvFTD and PD patients relative to HCs. To test this, FER performance was compared between groups via one-way ANOVAs, for each condition and emotion type. More specifically, we performed pairwise comparisons (HCs-bvFTD, HCs-PD, and HCs-AD) via Tukey's HSD tests, to assess specific interoceptive-emotional patterns in each patient group relative to HCs. Effect sizes were reported with Cohen's *d* and partial η squared (ηp^2^), as required. All FER behavioral analyses were performed using Pandas package (version 0.25.1) (McKinney, 2010), and Pingouin statistics package (version 0.3.6) (Vallat, 2018) in Python (version 3.7.4, Python Software Foundation), as well as BestNormalize package (Peterson and Cavanaugh, 2020) and lmerTest package (Kuznetsova et al., 2017) in R software (version 4.0.2, R Foundation for Statistical Computing). Figures were generated using the Seaborn Python package (version 0.9.0) (Batsis et al., 2019).

**Table 8.** Shapiro–Wilk test for normality

	HCs	bvFTD	PD	AD
FER phase negative emotions				
Post-Intero	*W* = 0.907, *p* < 0.001	*W* = 0.784, *p* < 0.001	*W* = 0.780, *p* < 0.001	*W* = 0.907, *p* = 0.04
Post-Extero	*W* = 0.889, *p* < 0.001	*W* = 0.890, *p* = 0.03	*W* = 0.877, *p* = 0.006	*W* = 0.936, *p* = 0.16
FER phase positive emotions				
Post-Intero	*W* = 0.916, *p* = 0.002	*W* = 0.914, *p* = 0.11	*W* = 0.670, *p* < 0.001	*W* = 0.961, *p* = 0.51
Post-Extero	*W* = 0.748, *p* < 0.001	*W* = 0.859, *p* = 0.009	*W* = 0.835, *p* = 0.001	*W* = 0.896, *p* = 0.02
FER phase neutral emotions				
Post-Intero	*W* = 0.879, *p* = 0.01	*W* = 0.810, *p* = 0.001	*W* = 0.877, *p* = 0.006	*W* = 0.879, *p* = 0.01
Post-Extero	*W* = 0.827, *p* < 0.001	*W* = 0.885, *p* = 0.03	*W* = 0.908, *p* = 0.02	*W* = 0.972, *p* = 0.78

#### hd-EEG preprocessing and analysis

Data were resampled offline at 256 Hz and filtered at 0.5-30 μV. Eye movements or blink artifacts were corrected with independent component analysis (D. Kim and Kim, 2012) and with a visual inspection protocol (Schandry and Montoya, 1996; Dirlich et al., 1997; Pollatos and Schandry, 2004; Terhaar et al., 2012; García-Cordero et al., 2016, 2017; Yoris et al., 2017, 2018; Salamone et al., 2018). R-wave values from the ECG signal were identified with a peakfinder function on MATLAB and used to segment continuous hd-EEG data for HEP analysis (Canales-Johnson et al., 2015; García-Cordero et al., 2016, 2017; Yoris et al., 2017; Salamone et al., 2018, 2020; Abrevaya et al., 2020; Legaz et al., 2020; Richter and Ibáñez, 2021). To examine the HEP occurring during the facial emotion stimuli presentation, we first segmented continuous EEG into epochs from −200 to 800 ms relative to the onset of facial stimuli (= 0). Then, within this 1000 ms window, the HEP was extracted for each condition. These EEG epochs were delimited between −300 and 500 ms around the R-wave peak, and baseline-corrected relative to −300 to 0 ms (Salamone et al., 2018). Epoch selection was designed to guarantee one full HEP modulation after each emotional stimulus. The signal was rereferenced offline to the average reference. Noisy epochs were rejected using an automatic EEGLAB procedure; criteria for exclusion included elimination of trials which exceeded a threshold of 2.5 SD from the mean probability distribution calculated from all trials and by measuring the kurtosis of probability distribution (Zich et al., 2015), percentage of rejected trails was similar across groups and conditions (for details, see Table 9). Low drifts were removed by linear trend corrections (Delorme and Makeig, 2004). HEP was then extracted for each priming condition (FER post-Intero or post-Extero) and for each stimuli valence (negative and neutral faces), and trials were averaged across subjects for group comparisons.

**Table 9.** EEG: percentage of rejected trails per condition*^a^*

Variable	HCs	bvFTD	PD	AD	Statistical results	*Post hoc*
NEG-Intero	25.41 (7.03)	24.60 (9.00)	22.47 (8.94)	20.97 (8.92)	*F* = 1.80, *p* = 0.15, ηp^2^ = 0.04	HCs-bvFTD: *p* = 0.98 HCs-PD: *p* = 0.46 HCs-AD: *p* = 0.14
NEG-Extero	26.22 (9.14)	23.98 (9.08)	23.28 (12.66)	23.30 (10.03)	*F* = 0.69, *p* = 0.55, ηp^2^ = 0.01	HCs-bvFTD: *p* = 0.84 HCs-PD: *p* = 0.64 HCs-AD: *p* = 0.66
NEU-Intero	20.06 (9.41)	18.15 (10.68)	20.48 (10.92)	18.48 (12.53)	*F* = 0.28, *p* = 0.83, ηp^2^ = 0.008	HCs-bvFTD: *p* = 0.91 HCs-PD: *p* = 0.99 HCs-AD: *p* = 0.93
NEU-Extero	24.42 (11.95)	24.12 (13.99)	22.41 (11.53)	19.52 (11.99)	*F* = 0.90, *p* = 0.44, ηp^2^ = 0.02	HCs-bvFTD: *p* = 0.99 HCs-PD: *p* = 0.90 HCs-AD: *p* = 0.39

*^a^*Data are mean (SD). The percentage of rejected trials was assessed through ANOVAs. Effect sizes were calculated through ηp^2^. No significant differences were found. All HEP results in HCs and HEP mean difference results were based on a similar amount of trials per emotional valence faces (negative – neutral) and condition (Intero – Extero). NEG, Negative emotion; NEU, Neutral emotion.

To test for potential effects of interoceptive priming on HEP modulation during the FER phase as hypothesized in HCs, we compared the modulation between negative and neutral faces for each priming condition (Intero vs Extero) in this group. In line with our hypothesis and previous supporting evidence, analyses focused on negative faces (Straube and Miltner, 2011; Couto et al., 2015; Terasawa et al., 2015; Georgiou et al., 2018; Marshall et al., 2018; Mai et al., 2019). Comparisons of HEP modulations were performed using a 5000 point-by-point Monte Carlo permutation test with bootstrapping (Manly, 2006). This analysis constitutes a robust approach for HEP analyses (Couto et al., 2014, 2015; Canales-Johnson et al., 2015; García-Cordero et al., 2016, 2017; Yoris et al., 2017, 2018; Salamone et al., 2018, 2020; Abrevaya et al., 2020; Legaz et al., 2020; Richter and Ibáñez, 2021), providing a solution for the multiple comparison problems and circumventing Gaussian distribution assumptions (Nichols and Holmes, 2002). To avoid cardiac field artifacts (Kern et al., 2013), we only analyzed time points between 200 and 500 ms, as these time points are proposed to be less affected by cardiac field artifacts (Dirlich et al., 1997; Kern et al., 2013; Park et al., 2014), and they capture a typical HEP latency (Pollatos and Schandry, 2004; Canales-Johnson et al., 2015; Müller et al., 2015; Pollatos et al., 2016). The main HEP analyses were based on a frontal ROI associated with interoceptive attention modulation (Couto et al., 2014; García-Cordero et al., 2016; Marshall et al., 2017). The ROI was composed of 11 electrodes: C9, C10, C14, C15, C18, C19, C20, C27, C28, C31, and C32. Additional analyses with three frontal ROIs (left-frontal, central-frontal, and right-frontal ROIs) were performed to evaluate the modulation in different locations. To ensure that results were not driven by different numbers of trials in each condition (negative vs neutral), we performed a complementary analysis selecting equal number of trials for each condition. To do this, for each subject, a random number of negative trials was extracted that matched that subject's number of neutral trials. The function randperm on MATLAB was used to select a random list of trials without repetitions.

Then, to test whether the interoceptive priming of negative emotions involved cortical neurophysiological alterations in patients, we compared HCs' FER-related HEP modulations with those of each pathologic group. To this end, for each group, we calculated the mean HEP modulation of each emotion type and subtracted the HEP modulation of negative-minus-neutral faces in each condition (post-Intero and post-Extero), based on the significant time window established for HCs (232–251 and 290–317 ms; for details, see Results). Finally, we compared the obtained indexes in each HCs-patient group pair using a nonparametric test (Wilcoxon rank-sum *p* < 0.05).

#### MRI preprocessing and analysis

Preprocessing included removal of nonbrain tissue, an automatic Talairach transformation, segmentation of the subcortical white matter (WM) and deep GM volumetric structures (including hippocampus, amygdala, caudate, putamen, and ventricles), intensity normalization, tessellation of the GM-WM boundary, an automatic topology correction, and surface deformation following intensity gradients to optimally place the GM/WM and GM/CSF borders at the location where the greatest shift in intensity defines the transition to the other tissue class. All T1 images were processed via surface-based morphometry (SBM) on FreeSurfer software suite (version 6.0; https://surfer.nmr.mgh.harvard.edu/). Structural surface-based metrics included cortical volume and thickness. SBM avoids registration to a standard space, overcoming registration errors, improving parcellation, and offering reliable estimation of region-specific differences (Clarkson et al., 2011). Full details on the implemented methods can be found elsewhere (Fischl, 2012). The plain-text output of the FreeSurfer's pipeline was postprocessed on Python (version 3.7.4, Python Software Foundation) and transformed into a better structure for statistical analysis. To avoid potential biases because of differences among the participants' head size (Whitwell et al., 2001), volume measures of each area were normalized as a percentage of the estimated total intracranial volume (provided also in FreeSurfer's results). One HC and one AD patient had to be excluded from this analysis because of artifacts. Finally, we performed a site normalization to avoid MRI-setup-dependent bias in the measurements. For each center, volume of both HCs and patients was *z*-scored based on the mean and SD of the corresponding center's HCs (Donnelly-Kehoe et al., 2019).

We hypothesized that, across all groups, post-Intero-negative FER would correlate with the volume of key interoceptive-emotional regions (i.e., insula and the ACC). Therefore, we tested this hypothesis by assessing the association between cortical volume and FER outcomes (IES) on negative emotions after both tapping-priming conditions (Intero and Extero), through Spearman correlations with Pingouin statistics package (version 0.3.6) (Vallat, 2018) on Python (version 3.7.4, Python Software Foundation). Following previous procedures (Sollberger et al., 2009; García-Cordero et al., 2016; O'Callaghan et al., 2016) and considering the moderate size of our experimental samples, all four groups were included in the analysis to increase behavioral variance and statistical power. To target relevant areas, analyses were made on masks, including canonical interoceptive cortical regions (insula, rostral ACC, and postcentral) also involved in emotional processes (Adolfi et al., 2017). Statistical significance was set at *p* < 0.05, corrected via false discovery rate (FDR).

#### FC preprocessing and analysis

Based on the fMRI resting-state recordings, we evaluated the different patterns of positive association between FER outcomes for negative emotions (after both tapping-priming conditions: Intero and Extero) and FC patterns. Images were preprocessed on an open-access toolbox: the Data Processing Assistant for Resting-State fMRI (DPARSFv2.3) (Chao-Gan and Yu-Feng, 2010).

To ensure that magnetizaion achieved a steady state, we discarded the first five volumes of each subject's resting-state recording. Then, images were preprocessed using an open-access toolbox: DPARSFv2.3 (Chao-Gan and Yu-Feng, 2010), which generates an automatic pipeline for fMRI analysis by calling the Statistical Parametric Mapping software (SPM12) (http://www.fil.ion.ucl.ac.uk/spm/software/spm12/) and the Resting-State fMRI Data Analysis Toolkit (REST V.1.7). As in previous studies (Salamone et al., 2018; Yoris et al., 2018), preprocessing steps included slice-timing correction (using middle slice of each volume as the reference scan) and realignment to the first scan of the session to correct head movement (SPM functions) (García-Cordero et al., 2016; Melloni et al., 2016; Sedeño et al., 2017). Then, images were normalized to the MNI space using the default EPI template from SPM12, smoothed using an 8 mm FWHM isotropic Gaussian kernel, and bandpass filtered between 0.01 and 0.08 Hz to correct and remove low-frequency drifts from the MR scanner. Finally, we regressed out six motion parameters, CSF, and WM signals to reduce the effect of motion and physiological artifacts, such as cardiac and respiration effects (REST V1.7 toolbox). Motion parameters were estimated during realignment, and CSF and WM masks were derived from the tissue segmentation of each subject's T1 scan in native space with SPM12 (after coregistration of each subject's structural image with the functional image). Finally, we excluded recordings with movements >3 mm and/or rotation movements >3° (for details, see Extended Data Fig. 5-1).

We hypothesized that SN connectivity would correlate with post-Intero-negative FER across all groups, but not in bvFTD. Also, we predicted that EN connectivity would correlate with post-Extero-negative FER in all groups. Consequently, after preprocessing, we used seed analyses to examine associations between behavioral outcomes and FC along the SN, related to Intero and emotion (Uddin, 2015; Adolfi et al., 2017; J. Kim et al., 2019; Seeley, 2019) and the EN, implicated in exteroceptive processes (Sridharan et al., 2008). To test the specificity of our predictions for these networks, we also examined associations between performance and connectivity along three additional control networks: the default-mode network (DMN), the visual network (VN), and the motor network (MN). We expected null associations between both post-Intero and post-Extero and these three control networks. We placed two bilateral seeds on cubic ROIs with a size of 7 × 7 × 7 voxels (Koslov et al., 2011) for each network. Each pair of seeds was located on different coordinates to capture each network's connectivity: (1) the dorsal ACC, a main hub of the SN (Seeley et al., 2007) (MNI coordinates 10, 34, 24 and −10, 34, 24); (2) the right and left superior frontal gyri of the EN (Boord et al., 2017) (MNI coordinates 30, −2, 62 and −30, −2, 62); (3) the posterior cingulate cortex, a key node of the DMN (Uddin et al., 2009) (MNI coordinates 3,−54, 27 and −3,−54, 27); (4) the primary visual cortex for the VN (Saiote et al., 2016) (MNI coordinates 8, −92, 8 and −8, −92, 8); and (5) the primary motor cortex (M1) for the MN (Vahdat et al., 2011) (MNI coordinates 32, −30, 68 and 332, −30, 68). To calculate the resting-state networks, we used Pearson's correlation coefficient across the whole time series obtained in the resting-state acquisition. Then, we used standard masks (Shirer et al., 2012) to isolate the voxels that are typically involved in each resting-state network. Finally, we spatially averaged across all included voxels to obtain one feature per network. To increase behavioral variance and statistical power, we first performed analyses, including all four groups (HCs, bvFTD, PD, and AD), and then included both patients and controls in each analysis (bvFTD-HCs, PD-HCs, AD-HCs) (Sollberger et al., 2009; García-Cordero et al., 2016; O'Callaghan et al., 2016). Resulting connectivity maps were correlated with FER outcomes for negative emotions (after both tapping-priming conditions: Intero and Extero) through the SPM12 multiple regression module (FDR-corrected, *p* = 0.05).

#### Data availability

Anonymized data that support the study's findings are available from open-source software (Salamone and Legaz, 2021) or from the corresponding author on reasonable request. The task is available online at https://github.com/pausalamone/Interoception.

## Results

### Behavioral results

#### Tapping-priming phase: interoceptive results

Compared with HCs, bvFTD patients exhibited selective impairments of Intero. No significant difference was observed in AD or PD patients relative to HCs. Concerning exteroceptive outcomes, no significant differences appeared in any group of patients with respect to HCs (Table 10). These results confirmed a compromise of Intero only in bvFTD patients.

**Table 10.** Tapping-priming results*^a^*

Condition	HCs	Patient samples	Student's *t* test	*p*
Intero	1.02 (0.34)	bvFTD: 0.75 (0.69)	2.15	0.03*
		PD: 1.09 (0.37)	0.82	0.42
		AD: 0.90 (0.41)	1.28	0.20
Extero	1.33 (1.60)	bvFTD: 0.64 (1.54)	1.51	0.13
		PD: 0.58 (1.39)	1.87	0.06
		AD: 0.96 (1.32)	1.90	0.37

*^a^*Data are mean (SD).

*Statistically significant differences.

#### FER phase: effects of interoceptive priming

**Figure 2.**
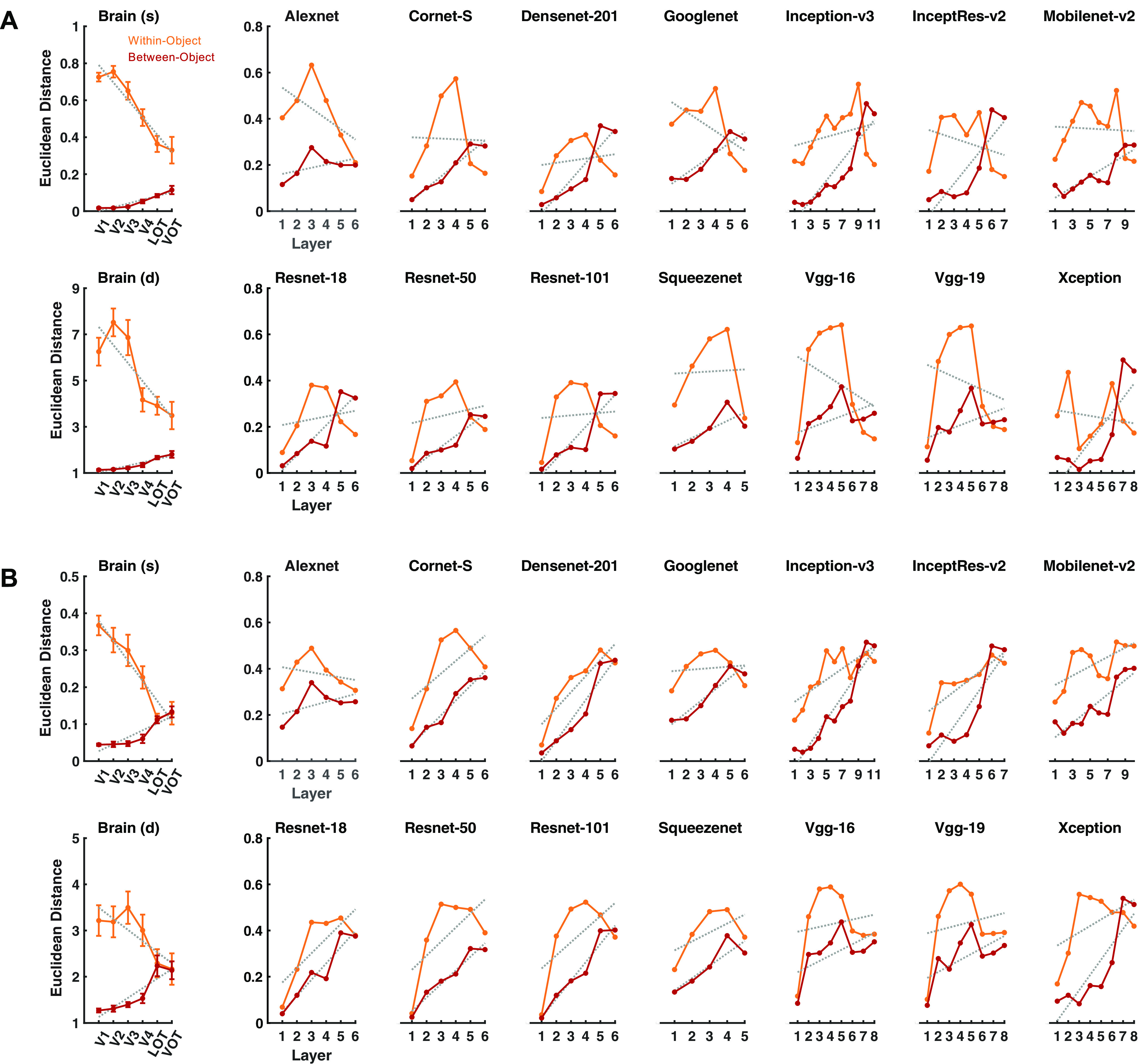
Positive and neutral FER in HCs. Results of positive and neutral FER comparing post-Intero and post-Extero effects in HCs. No interoceptive priming effects over FER were found for the two types of emotions: positive, *t* = 0.07, *p* = 0.94, Cohen's *d* = 0.01; neutral, *t* = −1.61, *p* = 0.11, Cohen's *d* = 0.20.

To corroborate the link between Intero and emotions, we examined whether interoceptive priming improved FER outcomes (normalized IES) in HCs. Relative to the post-Extero condition, post-Intero yielded better IES only for negative emotions (*t* = −2.63, *p* = 0.01, Cohen's *d* = 0.27). No differences were found for the other two types of emotion (positive: *t* = 0.07, *p* = 0.94, Cohen's *d* = 0.01; neutral: *t* = −1.61, *p* = 0.11, Cohen's *d* = 0.20) (Figs. 1*B*, 2). Briefly, HC exhibited the expected selective interoceptive (but not exteroceptive) priming of negative (but not other) emotions.

We also tested this priming effect in each emotion across patients compared with HCs. For negative emotions (Fig. 1*C*), we found significant differences between groups in post-Intero FER (*F*_(3,110)_ = 10.41, *p* < 0.001, ηp^2^ = 0.22). Specifically, *post hoc* comparisons demonstrated significantly lower negative emotion recognition post-Intero relative to HCs (mean = −0.48, SD = 0.67) in all patient samples (bvFTD: mean = 0.15, SD = 1.04, *p* = 0.02; PD: mean = 0.17, SD = 0.93, *p* = 0.007; AD: mean = 0.62, SD = 0.73, *p* < 0.001). In contrast, while significant differences were found between groups in post-Extero-negative FER (*F*_(3,108)_ = 8.37, *p* < 0.001, ηp^2^ = 0.18), *post hoc* comparisons revealed deficits only in the AD sample (AD: mean = 0.61, SD = 0.71, *p* < 0.001) relative to HCs (mean = −0.33, SD = 0.62). The other groups did not exhibit significant differences against HCs in post-Extero outcomes (bvFTD: mean = 0.19, SD = 1.05, *p* = 0.06; PD: mean = 0.11, SD = 0.76, *p* = 0.09).

For positive emotions, no significant group differences were found in post-Intero-positive FER (*F*_(3,110)_ = 2.42, *p* = 0.06, ηp^2^ = 0.06). In post-Extero-positive FER, although we found significant differences between groups (*F*_(3,109)_ = 2.83, *p* = 0.04, ηp^2^ = 0.07), only AD patients (mean = 0.92, SD = 1.00, *p* = 0.02) exhibited lower outcomes than HCs (mean = 0.09, SD = 1.15) in *post hoc* comparisons (Table 11).

**Table 11.** FER results*^a^*

Emotion	Priming	HCs	bvFTD	PD	AD	Statistical results	*Post hoc* comparisons
Negative	Post-Intero	−0.48 (0.67)	0.15 (1.04)	0.17 (0.93)	0.62 (0.73)	*F*_(3,110)_ = 10.41, *p* < 0.001*, ηp^2^ = 0.22	HCs-bvFTD: *p* = 0.02* HCs-PD: *p* = 0.007* HCs-AD: *p* < 0.001*
	Post-Extero	−0.33 (0.62)	0.19 (1.05)	0.11 (0.76)	0.61 (0.71)	*F*_(3,108)_ = 8.37, *p* < 0.001*, ηp^2^ = 0.18	HCs-bvFTD: *p* = 0.06 HCs-PD: *p* = 0.09 HCs-AD: *p* < 0.001*
Positive	Post-Intero	0.11 (1.05)	0.63 (1.25)	0.31 (1.14)	0.76 (0.73)	*F*_(3,110)_ = 2.42, *p* = 0.06, ηp^2^ = 0.06	HCs-bvFTD: *p* = 0.26 HCs-PD: *p* = 0.85 HCs-AD: *p* = 0.08
	Post-Extero	0.09 (1.15)	0.40 (1.07)	0.47 (1.14)	0.92 (1.00)	*F*_(3,109)_ = 2.83, *p* = 0.04*, ηp^2^ = 0.07	HCs-bvFTD: *p* = 0.73 HCs-PD: *p* = 0.52 HCs-AD: *p* = 0.02*
Neutral	Post-Intero	−0.74 (0.81)	−0.38 (0.95)	−0.39 (1.09)	0.18 (0.60)	*F*_(3,109)_ = 5.65, *p* = 0.001*, ηp^2^ = 0.13	HCs-bvFTD: *p* = 0.41 HCs-PD: *p* = 0.35 HCs-AD: *p* < 0.001*
	Post-Extero	−0.59 (0.72)	−0.15 (0.90)	−0.42 (0.82)	0.08 (0.73)	*F*_(3,107)_ = 4.12, *p* = 0.008*, ηp^2^ = 0.10	HCs-bvFTD: *p* = 0.18 HCs-PD: *p* = 0.81 HCs-AD: *p* < 0.007*

*^a^*Data are mean (SD). Between-group comparison on FER performance post-Intero and post-Extero was assessed through one-way ANOVAs and Tukey *post hoc* comparisons. Effect sizes were calculated through ηp^2^.

*Significant difference.

For neutral emotions, significant differences between groups were found in post-Intero (*F*_(3,109)_ = 5.65, *p* = 0.001, ηp^2^ = 0.13) and post-Extero (*F*_(3,107)_ = 4.12, *p* = 0.008, ηp^2^ = 0.10) neutral FER. *Post hoc* comparisons revealed significant deficits only in AD patients (Post-Intero: mean = 0.18, SD = 0.60, *p* < 0.001; Post-Extero: mean = 0.08, SD = 0.73, *p* = 0.007) compared with HCs (Post-Intero: mean = −0.74, SD = 0.81; Post-Extero: mean = −0.59, SD = 0.72) (Table 11).

### EEG results

#### HEP modulation during emotion recognition after priming

**Figure 3.**
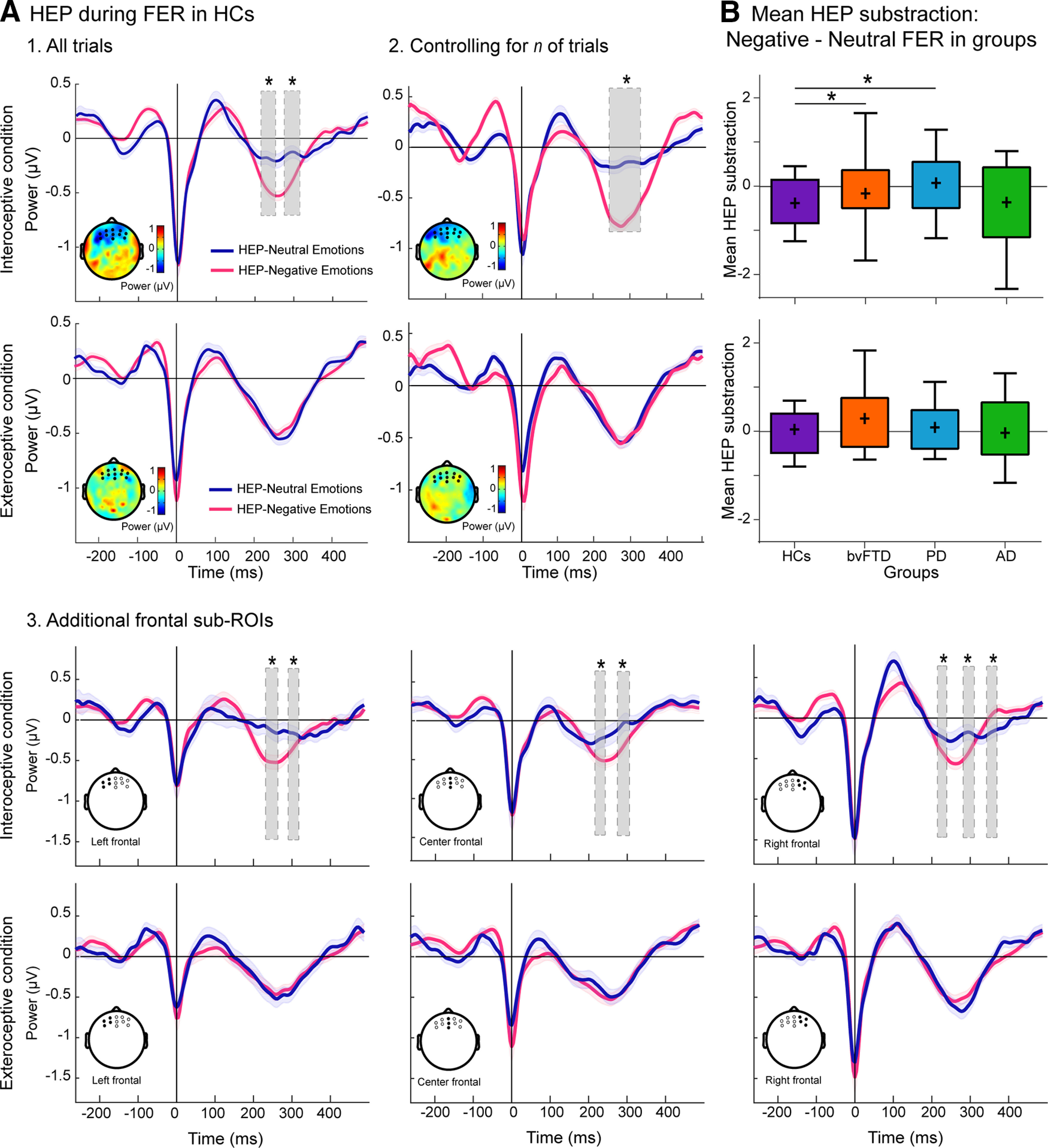
HEP during FER. ***A***, HEP during FER in HCs. ***A1***, All trials. HEP modulations after seeing negative (magenta line) versus neutral (dark blue line) emotional faces in HCs, over a frontal ROI, among all trials. Post-Intero and post-Extero condition comparisons are shown in the first and second row, respectively. Gray shaded boxes represent statistically significant differences at *p* < 0.05 for a minimum extension of five consecutive points of difference (Salamone et al., 2018) (from 232 to 251 ms, and from 290 to 317 ms). Scalp topographies represent the electrodes used for the frontal ROI and the differences in amplitude (microvolts) between ERPs at 240 ms. A wavelet-based method was used to smooth ERPs only for visualization purposes. Shadowed lines around the HEP indicate the SEM. These results were obtained with a demographically matched sample (Table 4). Results were based on similar amount of trials per emotional valence faces (negative – neutral) and condition (Intero – Extero) as detailed in Table 9. ***A2***, Controlling *n* of trials. Results after extracting a random subsample of trials for the negative condition, matching the number of trails in the neutral condition of each subject in HCs. Significant differences were found between negative-neutral HEP modulations in post-Intero. Scalp topographies show the electrodes used for the frontal ROI and the differences in amplitude (microvolts) between ERPs at 240 ms. ***A3***, Additional frontal sub-ROIs. Columns 1-3 represent results for left, middle, and right frontal sub-ROIs, respectively. First row represents post-Intero comparison. Second row represents post-Extero comparison. All frontal sub-ROIs in HCs showed significant differences between negative-neutral HEP modulations in post-Intero. On the contrary, no significant differences were found in post-Extero. ***B***, Subtraction of mean HEP modulation between negative and neutral faces. Subtraction between the mean difference of the HEP modulation between negative and neutral faces within the time points showed significant differences in the HC sample (***A***, gray area; all trials: interoceptive condition). Boxplot represents results for HCs (purple), bvFTD (orange), PD (light blue), and AD (green) participants, with the mean identified via a cross (+). Whiskers represent all data comprised between the 10th and 90th percentile. *p < 0.05, significant difference with the HC group.

To evaluate interoceptive priming effects on FER-related HEP modulations, we tested the differences between negative and neutral faces for each tapping-priming condition in HCs. In the post-Intero condition, HCs showed significantly larger HEP amplitudes for negative than neutral faces in two canonical windows (232-251 and 290-317 ms; Fig. 3*A*). No differences emerged in the post-Extero condition. Results were not driven by a different number of trials between negative and neutral conditions (Fig. 3*A*). Moreover, these results were replicated in the additional frontal sub-ROIs (Fig. 3*A*).

Based on the time window detected in HCs, we compared the mean HEP modulation for negative minus neutral faces across groups. In the post-Intero condition, the negative-neutral HEP difference was higher for HCs than bvFTD (*W* = 251, *p* = 0.02, Cohen's *d* = 0.33) and PD (*W* = 348, *p* = 0.03, Cohen's *d* = −0.48) patients, but similar to that of AD patients (*W* = 413, *p* = 0.18, Cohen's *d* = 0.26) (Fig. 3*B*). The post-Extero condition yielded no significant differences between HCs and any patient group (bvFTD: *W* = 367, *p* = 0.64, Cohen's *d* = 0.16; PD: *W* = 448, *p* = 0.91, Cohen's *d* = −0.08; AD: *W* = 346, *p* = 0.40, Cohen's *d* = −0.39).

### Neuroimaging results

#### Structural associations with negative FER

**Figure 4.**
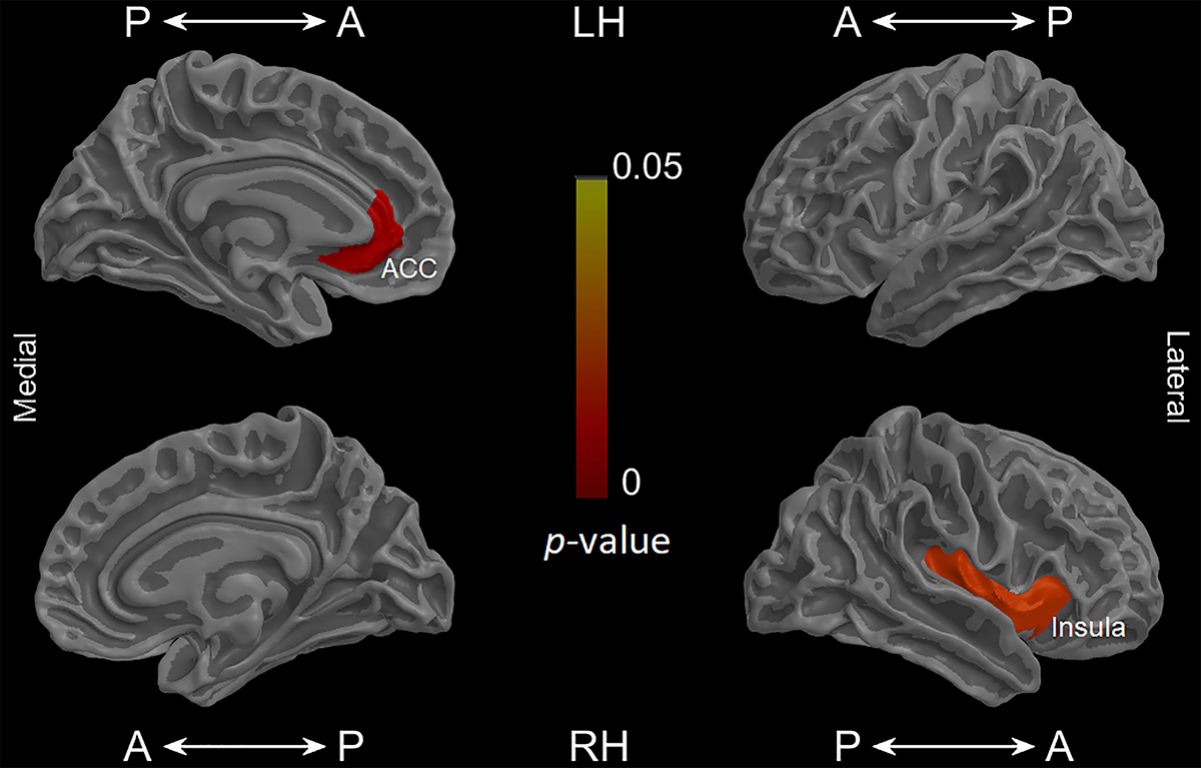
Associations between post-Intero-negative FER and cortical volume. Whole-group analyses revealed associations between post-Intero-negative FER (IES) and the cortical volume of canonical interoceptive-emotional regions (*p* < 0.05, FDR-corrected). For methodological details, see Table 5. Cortical volumes were obtained via SBM. Results are presented using Desikan-Killiany cortical atlas (Desikan et al., 2006) and obtained with a demographically matched sample (see Table 6). For GM atrophy patterns in patients, see Table 2. For structural association details, see Table 12. A, Anterior; LH, left hemisphere; P, posterior; RH, right hemisphere.

Analyses for all groups revealed significant associations (*p* < 0.05, FDR-corrected) between post-Intero-negative FER and the volume of emotional-interoceptive regions (right insula, left rostral ACC). No significant associations were found for post-Extero-negative FER (Fig. 4; Table 12).

**Table 12.** Association between cortical volume and post-Intero-negative FER*^a^*

Region	*R*	*p* (FDR)
All groups		
Insula R	−0.268	0.023*
Insula L	−0.209	0.059
Rostral anterior cingulate R	−0.225	0.053
Rostral anterior cingulate L	−0.311	0.011*
Postcentral R	−0.195	0.066
Postcentral L	−0.139	0.174

*^a^*Cortical volume measures were obtained through SBM. Spearman correlations were examined between cortical volume of main interoceptive/emotion areas and negative FER post-Intero outcomes, applying *p* < 0.05 with FDR correction. Results are presented using Desikan-Killiany cortical atlas (Desikan et al., 2006). No significant associations were found between cortical volume and post-Extero IES in negative emotions. R, Right; L, left.

*Significant association.

#### FC associations with negative FER

**Figure 5.**
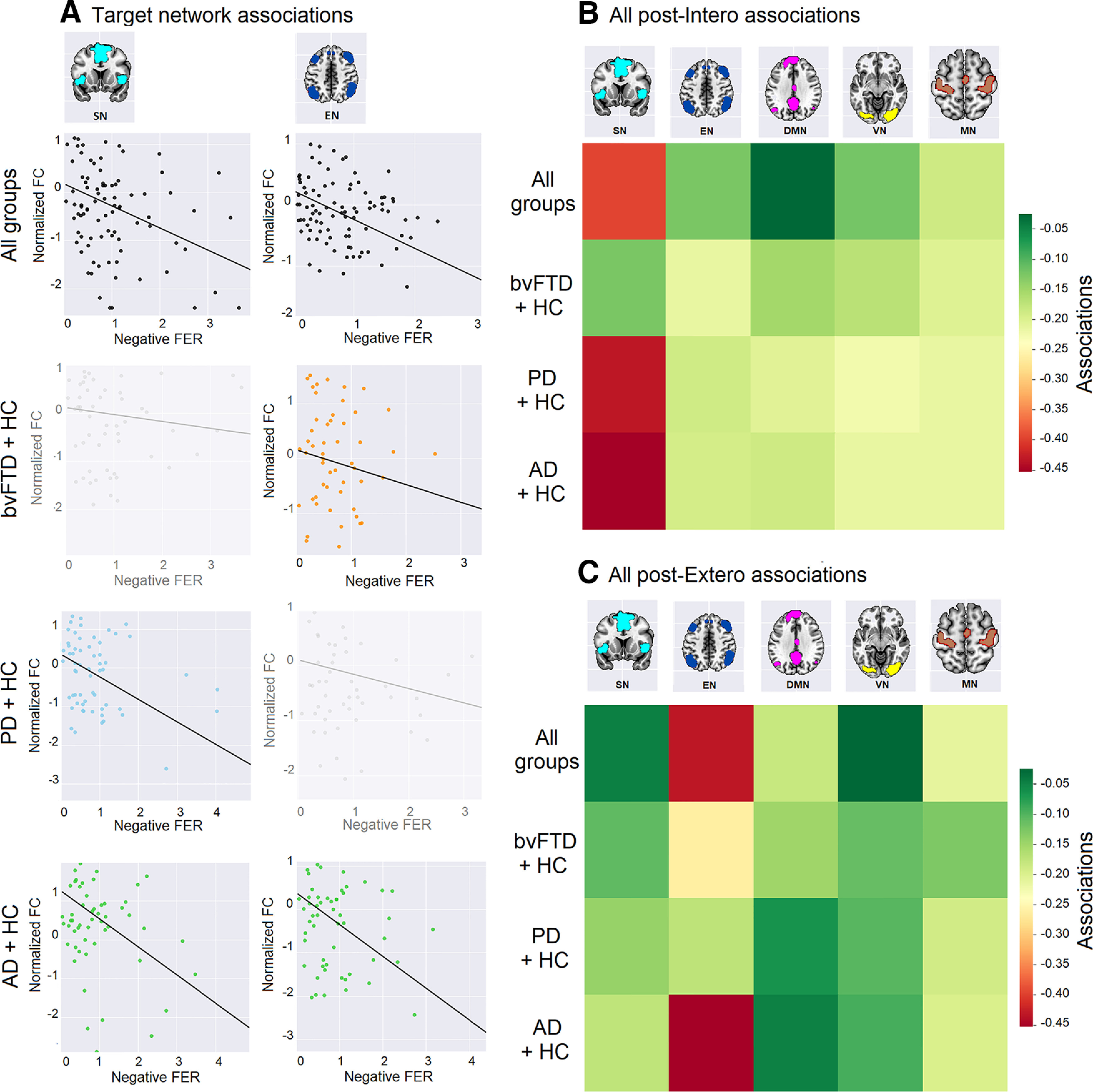
Associations between FC and negative FER. Seed analyses over five networks (SN, EN, MN, VN, DMN, *p* = 0.05 FDR-corrected) were performed to test the association between the FC of each network and FER outcomes for negative emotions (after both tapping-priming conditions: Intero and Extero), in all groups together and in tandems (bvFTD-HCs, PD-HCs, AD-HCs). ***A***, Target network associations. Associations are plotted between target networks (SN and EN) and outcomes in the corresponding negative FER phase (post-Intero and post-Extero, respectively). No significant associations were found between SN and post-Intero-negative FER in bvFTD, and between EN and post-Extero-negative FER in PD. All correlations between every other combination of network and condition were nonsignificant across groups. ***B***, All post-Intero associations. Correlation matrix between post-Intero among all groups and tandems, and networks. ***C***, All post-Extero associations. Correlation matrix between post-Extero among all groups and tandems, and networks. These results were obtained with a demographically matched sample (see Table 7). For FC details, see Table 13. Standard masks (Shirer et al., 2012) were used to isolate the voxels that are typically involved in each resting-state network, based on the MNI anatomic space. None of the participants showed head movements >3 mm and/or rotations >3° (see Extended Data Figure 5-1).

For all groups (Fig. 5), seed analyses revealed significant negative associations (i.e., the better the performance, the higher the FC) between post-Intero FER and SN (*r* = −0.39, *p*-FDR = 0.009), whereas post-Extero FER was significantly associated to EN (*r* = −0.57, *p*-FDR = 0.007) (Table 13). No associations were significant with the control networks (DMN, VN, MN).

10.1523/JNEUROSCI.2578-20.2021.f5-1 Figure 5-1 fMRI movement's parameters. Data are mean (SD) [range]. None of the included participants showed head movements greater than 3 mm and/or rotations higher than 3°Supekar et al., 2008. Download Figure 5-1, DOCX file.

**Table 13.** Association between FC and negative FER*^a^*

Network	Post-Intero		Post-Extero	
	*R*	*p* (FDR)	*R*	*p* (FDR)
All groups				
SN	−0.395	0.009*	−0.052	0.114
EN	−0.125	0.071	−0.571	0.007*
DMN	−0.025	0.125	−0.225	0.061
VN	−0.121	0.069	−0.025	0.125
MN	−0.187	0.095	−0.263	0.06
bvFTD + HCs				
SN	−0.125	0.071	−0.131	0.07
EN	−0.214	0.084	−0.328	0.014*
DMN	−0.152	0.076	−0.184	0.094
VN	−0.169	0.072	−0.135	0.085
MN	−0.202	0.061	−0.157	0.075
PD + HCs				
SN	−0.453	0.008*	−0.175	0.093
EN	−0.187	0.07	−0.214	0.069
DMN	−0.204	0.062	−0.075	0.106
VN	−0.225	0.061	−0.124	0.071
MN	−0.214	0.08	−0.235	0.068
AD + HCs				
SN	−0.432	0.008*	−0.216	0.069
EN	−0.192	0.093	−0.412	0.008*
DMN	−0.186	0.095	−0.054	0.113
VN	−0.215	0.069	−0.112	0.07
MN	−0.215	0.069	−0.245	0.064

*^a^*Seed analyses over five networks (SN, EN, MN, VN, DMN, *p* = 0.05 FDR-corrected) were performed to test the association between the FC of each network and post-Intero- and post-Extero-negative FER outcomes, in all groups together and in tandems (bvFTD-HCs, PD-HCs, AD-HCs). Significant associations were found between SN and post-Intero-negative FER in all subjects, PD and AD, and between EN and post-Extero-negative FER in all subjects, bvFTD, and AD.

*Significant difference.

In the bvFTD-HCs tandem, no network was associated with post-Intero performance. However, post-Extero FER of negative emotions was significantly associated with FC in the EN (*r* = −0.32, *p*-FDR = 0.014). The opposite pattern was found in the PD-HCs tandem, with significant associations between post-Intero-negative FER and FC in the SN (*r* = −0.45, *p*-FDR = 0.008), and no significant associations between FC and post-Extero performance. Finally, the AD-HC tandem presented significant associations between FC in SN and post-Intero FER (*r* = −0.43, *p*-FDR = 0.008), and between FC in EN and post-Extero FER (*r* = −0.41, *p*-FDR = 0.008).

## Discussion

We investigated the multimodal impact of Intero on negative emotions and its manifestation across neurodegenerative conditions and HCs. Behaviorally, interoceptive accuracy impairments were only observed in bvFTD. Intero primed negative FER in HCs and AD (in despite of generalized FER alterations in the latter group), but this effect was disrupted in bvFTD and PD patients. Increased HEP modulations during post-Intero-negative FER were present in HCs and AD, but not in bvFTD and PD. Anatomofunctional signatures in all groups evidenced the involvement of canonical cortical hubs (ACC and insula) and networks (SN) in the integration of interoceptive-emotional processes, and the role of the EN in exteroceptive emotional processes. Disrupted connectivity associations were found between post-Intero-negative FER and SN in bvFTD, and between post-Extero-negative FER and EN in PD, but both networks' associations were preserved in AD. These findings indicate that multimodal signatures of interoceptive priming are compromised in bvFTD, further revealing dynamic interactions between neurocognitive disruptions isolatedly acknowledged in affective (Rosen et al., 2004; Fernandez-Duque and Black, 2005; Kipps et al., 2009; Piguet et al., 2011; Kumfor et al., 2013) and interoceptive (García-Cordero et al., 2016; Van den Stock and Kumfor, 2017; Salvato et al., 2018; Ibáñez, 2019) clinical research.

### Behavioral effects of interoceptive priming on emotion recognition

We found a behavioral effect of interoceptive priming over negative FER in HCs, as expected from previous literature (Terasawa et al., 2014; Critchley and Garfinkel, 2017). Compared with HCs, bvFTD patients were the only group to present selective interoceptive deficits (with preserved Extero), accompanied by specific impairments in negative FER post-Intero. These results suggest that alterations in interoceptive predictive coding mechanisms are tied to abnormal emotional processing (Seth et al., 2011), consistent with recent theoretical proposals for this disease (Ibáñez et al., 2017; Van den Stock and Kumfor, 2017; Ibáñez, 2019). Negative FER impairments in PD replicate previous findings (Narme et al., 2011; Enrici et al., 2015; Argaud et al., 2018). As expected in AD, generalized FER deficits (Klein-Koerkamp et al., 2012) were observed even with preserved interoceptive processing. Together, behavioral results show three distinct patterns of patient deficit, with only bvFTD evidencing a specific deficit of interoceptive priors on FER.

### Electrophysiological markers of interoceptive priors

Electrophysiological signatures of selective interoceptive priming of negative emotions were observed in HCs, as expected (Couto et al., 2015). In comparison, aligned with the interpretation of emotionally compromised interoceptive coding (Seth et al., 2011), bvFTD patients did not present increased HEP modulation during negative FER. Although interoceptive accuracy was preserved in PD, no HEP modulation post-Intero was observed, suggesting interrupted ongoing links between Intero and emotion. Temporal estimation deficits in PD would be compatible with the hypothesis of impaired Bayesian temporal interoceptive inference (Adams et al., 2016; Seth and Friston, 2016). In AD (as in HCs), HEP modulations during post-Intero FER were preserved. Other general pathophysiological mechanisms (e.g., general cognitive impairment, mood disorders) (Shany-Ur and Rankin, 2011; Henry et al., 2016; Christidi et al., 2018) could explain these emotional processing deficits in AD. Briefly, relative to HCs, results show abolished electrophysiological markers of interoceptive priming (HEP) on FER across bvFTD and PD, with preserved patterns in AD.

### Anatomofunctional correlates on interoceptive-emotional integration

Our study also reveals anatomofunctional correlates of interoceptive-emotional blending, confirming previous metanalytic evidence of their neurocognitive overlaps (Adolfi et al., 2017). In all-group analyses, key cortical regions (insula, ACC) in addition to the SN were associated to post-Intero recognition of negative emotions. Complementary, the EN was associated to post-Extero performance. The SN processes interoceptive and emotional signals (Chong et al., 2017; Icenhour et al., 2017; Toller et al., 2018; J. Kim et al., 2019; Ruiz-Rizzo et al., 2020) and the EN guides appropriate responses to external stimuli (Uddin, 2015). Our findings implicate the SN in the integration of interoceptive and emotional processes, and the EN in exteroceptive emotional operations (Sridharan et al., 2008; Lindquist and Barrett, 2012). Moreover, no associations were found in the DMN, VN, or MN, confirming the specific impact of interoceptive and exteroceptive priming at network level. The SN association was specifically affected in bvFTD (Day et al., 2013; Seeley, 2019), suggesting that interoceptive networks do not modulate emotional processing in this disease, potentially contributing to patients' inability to adjust to social situations (Zhou and Seeley, 2014). In PD, the SN was preserved in post-Intero, suggesting that the behavioral and ongoing HEP deficits do not rely on this network and would constitute less specific deficits (i.e., temporal estimation deficits). Also, the association between post-Extero with the EN confirms the role of executive functions in emotional processing in PD (Péron et al., 2012; Moonen et al., 2017; Argaud et al., 2018). Thus, both results suggest unspecific interoceptive deficits that are not linked to multimodal mechanisms and cognitive processes, which are critical for emotional processing in PD. The AD group presented preserved FC modulations; and together with the conserved HEP modulations, these results suggest partially unaffected interoceptive priming mechanisms. These neurofunctional results converge in showing a distinctive altered pattern in bvFTD, unspecific associations in PD, and partially preserved predictive interoceptive coding in AD.

### Theoretical and clinical implications

These results carry theoretical and clinical implications. Our findings may support theoretical accounts of functional synergies between both domains (Garfinkel and Critchley, 2016; Seth and Friston, 2016; Owens et al., 2018; Pace-Schott et al., 2019), supporting models of cognitive blending (Ibáñez and Manes, 2012; Baez et al., 2017; Ibáñez et al., 2017; Ibáñez, 2018, 2019; Ibáñez and García, 2018; Ibáñez and Schulte, 2020). From an embodied perspective, since emotional processing is partly modulated by the perception on visceral information, understanding the integration of these multimodal signals could prove critical for patients' successful allostasis; that is, efficient preparation of physiological need and internal states relative to the context (Sterling, 2014; Kleckner et al., 2017). From a clinical perspective, a novel agenda is opened, related to basic and clinical innovations at the crossing of cognitive neuroscience and behavioral neurology. Present findings offer new insights about interventions related to body awareness (Fustos et al., 2013; Müller et al., 2015) and metacognition of emotion (García-Cordero et al., 2021), interoceptive priors, and allostatic predictive coding addressing patients' emotional-self regulation.

### Limitations and further research

Our study featured important limitations, calling for further research. First, our design was based on modest sample sizes. Nevertheless, these are similar to or larger than those of other multimodal reports assessing neurodegenerative subtypes (Moretti et al., 2009; Hughes et al., 2011; Premi et al., 2014; García-Cordero et al., 2016; Melloni et al., 2016). Also, this caveat was counteracted by the strict control clinical variables, as well as detailed diagnostic procedures and systematic assessments. Moreover, although our findings provide a multimodal picture across behavioral, electrophysiological, anatomic, and FC dimensions with moderate to large effect sizes, future studies should contemplate larger samples and alternative designs allowing for exploration of relevant effects and patient-group interactions outside the scope of our study.

Also, since the task required a tripartite (positive/neutral/negative) categorization (Rosen et al., 2004; Fernandez-Duque and Black, 2005; Kipps et al., 2009), and considering the relatively low number of trials per emotion and the need guarantee adequate signal-to noise ratios for EEG data, different negative emotions (anger, disgust, fear, sadness) were not assessed individually. Moreover, the valence of “surprise” may prove ambiguous, prompting both positive and negative interpretations (M. J. Kim et al., 2017, 2020; Petro et al., 2018). Future tasks could tackle these shortcomings and provide more fine-grained results for each emotion individually. In addition, new studies could also examine both short- and long-lasting HEP effects, by analyzing several HEP using longer periods of stimulus presentation and interstimulus intervals.

Finally, by targeting a multimodal neurodegenerative lesion model (Rorden and Karnath, 2004; García-Cordero et al., 2016; Melloni et al., 2016), we provided evidence on the relationship of Intero and emotion. This approach may be expanded with direct stimulation techniques (vagal peripheral stimulation, electrical stimulation with intracranial recordings) to assess how the modulation of visceral or neural signals underlying Intero impact of emotional processing outcomes.

In conclusion, research on the interactions between internal body signals and emotion has a long tradition in cognitive neuroscience, neurology, and related disciplines. Our multimodal, cross-pathologic approach reveals novel links between Intero and emotion recognition, pointing to its distinct disruptions in bvFTD, unspecific deficits in PD, and preserved interoceptive priming effects, despite generalized FER deficits in AD. These results may support theoretical accounts of functional synergies between both domains while opening a novel agenda for basic and clinical innovations at the crossing of cognitive neuroscience and behavioral neurology.
