# Supplementary material for: Interoception Primes Emotional Processing: Multimodal Evidence from Neurodegeneration
Source: J Neurosci. 2021 May 12;41(19):4276–92. doi: 10.1523/JNEUROSCI.2578-20.2021 (PMC8143206; doi:10.1523/JNEUROSCI.2578-20.2021)
Supplement: Figure 5-1 — fMRI movement's parameters. Data are mean (SD) [range]. None of the included participants showed head movements greater than 3 mm and/or rotations higher than 3°Supekar et al., 2008. Download Figure 5-1, DOCX file. [file ns-JN-RM-2578-20-s01.docx]

**Figure 5-1. fMRI movement’s parameters**

| **Movement’s parameters** | | | | |
| --- | --- | --- | --- | --- |
| **Variable** | **HCs** | **bvFTD** | **PD** | **AD** |
| Translation (mm) | 0.09 (0.06)  [0.02 – 0.26] | 0.08 (0.05)  [0.02 – 0.22] | 0.08 (0.04)  [0.02 – 0.15] | 0.09 (0.04)  [0.03 – 0.18] |
| Rotation (º) | 0.05 (0.03)  [0.01 – 0.19] | 0.03 (0.05)  [0.01 – 0.11] | 0.06 (0.06)  [0.02 – 0.22] | 0.07 (0.04)  [0.02 – 0.18] |

Results are presented as mean (*SD*) [range]. None of the included participants showed head movements greater than 3 mm and/or rotations higher than 3º (Supekar et al., 2008). HCs: healthy controls; bvFTD: behavioral variant frontotemporal dementia; PD: Parkinson’s disease; AD: Alzheimer’s disease.
